# Hypocotyl adventitious root organogenesis differs from lateral root development

**DOI:** 10.3389/fpls.2014.00495

**Published:** 2014-09-29

**Authors:** Inge Verstraeten, Sébastien Schotte, Danny Geelen

**Affiliations:** Department of Plant Production, Faculty of Bioscience Engineering, Ghent UniversityGhent, Belgium

**Keywords:** adventitious root, lateral root, differential regulation, plant growth regulators, *Arabidopsis thaliana*

## Abstract

Wound-induced adventitious root (AR) formation is a requirement for plant survival upon root damage inflicted by pathogen attack, but also during the regeneration of plant stem cuttings for clonal propagation of elite plant varieties. Yet, adventitious rooting also takes place without wounding. This happens for example in etiolated *Arabidopsis thaliana* hypocotyls, in which AR initiate upon de-etiolation or in tomato seedlings, in which AR initiate upon flooding or high water availability. In the hypocotyl AR originate from a cell layer reminiscent to the pericycle in the primary root (PR) and the initiated AR share histological and developmental characteristics with lateral roots (LRs). In contrast to the PR however, the hypocotyl is a determinate structure with an established final number of cells. This points to differences between the induction of hypocotyl AR and LR on the PR, as the latter grows indeterminately. The induction of AR on the hypocotyl takes place in environmental conditions that differ from those that control LR formation. Hence, AR formation depends on differentially regulated gene products. Similarly to AR induction in stem cuttings, the capacity to induce hypocotyl AR is genotype-dependent and the plant growth regulator auxin is a key regulator controlling the rooting response. The hormones cytokinins, ethylene, jasmonic acid, and strigolactones in general reduce the root-inducing capacity. The involvement of this many regulators indicates that a tight control and fine-tuning of the initiation and emergence of AR exists. Recently, several genetic factors, specific to hypocotyl adventitious rooting in *A. thaliana*, have been uncovered. These factors reveal a dedicated signaling network that drives AR formation in the *Arabidopsis* hypocotyl. Here we provide an overview of the environmental and genetic factors controlling hypocotyl-born AR and we summarize how AR formation and the regulating factors of this organogenesis are distinct from LR induction.

## INTRODUCTION

Plant roots, hidden below the soil surface, are generally overlooked when one thinks about plant growth and development (Smith and De Smet, 2012). Roots however, are vital for plant growth and function in the uptake of water and nutrients and anchor the plant in its environment (Petricka et al., 2012). The blue print for the primary root (PR) is established during embryogenesis (Scheres et al., 1994), but most root organs are formed post-embryonically. This enables a plant to shape the root architecture in accordance to its needs and the physical constraints imposed by its environment (Hodge et al., 2009; Den Herder et al., 2010; Lynch and Brown, 2012; Lavenus et al., 2013). Monitoring environmental conditions and subsequent translation into signaling processes is key to develop a well-adapted root system (Rymen and Sugimoto, 2012). Due to the importance of roots to ensure crop yield under suboptimal water-availability or low soil fertility, the plant’s root system is an important target trait to improve crop performance (Lynch, 2007; Kell, 2011; Jung and McCouch, 2013). It therefore is relevant to acquire further insights into root formation, root architecture and the controlling physiological and genetic factors.

The plasticity of the plant’s morphology results in a variable root entity, consisting of different root-types organized in a varying spatial configuration. In general, the root system is comprised of at least two root-types: the PR, which is formed during embryogenesis, and post-embryonically initiated lateral roots (LRs) branching of the PR (Celenza et al., 1995). In addition to LR, plants can develop adventitious roots (ARs), which emerge from non-root tissues, such as stems and leaves (Geiss et al., 2009). Some plant species spontaneously develop AR during vegetative reproduction. In other species, AR are specialized organs with specific properties, for example air supply, which is the case for pneumatophoric AR in epiphytic orchids, or support, which occurs in ivy or in stilt roots in mangroves. Moreover, the crown roots, a specific type of AR initiating on predestined nodes in the stems of monocotyledonous plants (e.g., rice and corn), constitute the main roots of monocot root systems (Hochholdinger et al., 2004; Osmont et al., 2007; Bellini et al., 2014). Despite the potential access to unlimited resources via undetermined root growth, plants have limited endogenous energy reserves. Therefore, a strict control of their investment in either primary or secondary roots is necessary. This implicates the existence of a tight regulation of different hormonal signaling pathways and factors regulating root development.

The flexible adaptation of the root system to heterogeneous soil environments, the response to nutrients or water availability and the complex hormonal control of root organogenesis provide significant scientific challenges (Monshausen and Gilroy, 2009). Additionally, the variable constitution of a plant’s root system, with a complex mix of different root-types complicates the deciphering of the importance of each individual root-type. Research in a model species could aid the root-type-specific analysis. Therefore, a series of studies in the model plant *Arabidopsis thaliana* has been undertaken (Casson and Lindsey, 2003; Lavenus et al., 2013). Much attention has been given to the development of the PR and to LR initiation, largely because of the amenability of the *Arabidopsis* root system to microscopic observation and genetic dissection (Beeckman et al., 2001; Casimiro et al., 2001, 2003; Himanen et al., 2002, 2004; De Smet et al., 2006a; Péret et al., 2009a,b; Lavenus et al., 2013). However, etiolated seedlings of this species also develop AR on the elongated hypocotyl and auxin treatments of inflorescence stem segments or leaves result in AR development (Ludwig-Müller et al., 2005; da Rocha Corrêa et al., 2012a,b; da Costa et al., 2013; Verstraeten et al., 2013). Moreover also in light grown seedlings, without an elongated hypocotyl, AR are initiated on the hypocotyl upon excision of the PR (Sukumar et al., 2013). The spontaneous and induced formation of AR in *A. thaliana* demonstrates that AR are an integral part of the root system. The phenomenon of AR induction can therefore successfully be studied in this species. In addition to *Arabidopsis*, other species such as tomato, mung bean, pine and sunflower, develop AR on the hypocotyl and have been used in research. In this review we focus on recently acquired knowledge about AR formation and the different regulating factors of primary and secondary root development. In this respect, the hypocotyl is our tissue of interest as it shares similarities, such as the presence of a meristematic pericycle cell layer, which gives rise to LR or AR, and differences to the PR, such as the signaling process leading to AR initiation which differs from the processes leading to LR development. We provide an overview of the environmental, physiological and genetic determinants for the diverse root-types and we summarize how their organogenesis is fundamentally different.

## DIFFERENT ROOT-TYPES IN *Arabidopsis thaliana*

### PRIMARY ROOT

The basic feature of a polar plant body, with an aerial part and a PR is already present in the embryo. Upon germination, the PR emerges from the seed and grows gravitropically into the soil by cellular divisions in the apical meristem and subsequent cell elongation (Beemster and Baskin, 1998). The existence of a meristematic center at the root tip is of importance to maintain the root growth. This meristem acts as a growth-organizing center and consists of quiescent cells (QCs), which remain undifferentiated, surrounded by initial cells. These initials differentiate to specific cell types upon asymmetrical divisions (Dolan et al., 1993; Sabatini et al., 2003; Garay-Arroyo et al., 2012). The coordinated divisions of the initial cells lead to a radial pattern of concentric cell layers, each with specific functions (Scheres et al., 2002). Due to the radial organization and the generation of new cells by divisions at the tip, followed by cell elongation and differentiation, spatio-temporal transcript maps of each root cell type are generated (Birnbaum et al., 2003). Some of the key molecular regulators of the PR meristem, such as PLT1 (*PLETHORA1*), SCR (*SCARECROW*), and SHR (*SHORT ROOT*) are also involved in the maintenance of the LR stem cell niche, and most likely also in the AR meristem (Lucas et al., 2010; Della Rovere et al., 2013; Tian et al., 2014). We therefore don’t discuss these factors in this review.

Endogenous auxin accumulates in the root tip where it coordinates cell divisions, cell expansion and contributes to the gravitropic response of the root (Sabatini et al., 1999; Petersson et al., 2009; Overvoorde et al., 2010). High levels of auxin stimulate cell division, whereas reduced levels mainly stimulate elongation (Perrot-Rechenmann, 2010). Besides the concentration, also the auxin-type is important to determine cell fate. For example, exogenous 1-naphthaleneacetic acid stimulates cell elongation, while 2,4-dichlorophenoxyacetic acid induces divisions (Campanoni and Nick, 2005). Other plant growth regulators also affect PR growth. Cytokinins for example, determine the rate of cell divisions in the PR meristem, potentially via a redistribution of auxin (Dello Ioio et al., 2007; Chapman and Estelle, 2009; Růžička et al., 2009; Ubeda-Tomás et al., 2012; Della Rovere et al., 2013; Zhang et al., 2013). Abscisic acid (ABA) on the other hand, inhibits cell cycle progression (Wang et al., 1998). Gibberellins (GAs) control the transition to cell elongation (Achard et al., 2009; Ubeda-Tomás et al., 2009). Moreover, hormone actions in the PR often depend on the interaction with auxin biosynthesis and transport (Mähönen et al., 2006; Růžička et al., 2007; Swarup et al., 2007; Bishopp et al., 2011a,b; Liu et al., 2014). It can therefore be concluded that different plant hormone signaling pathways converge at the control of cell divisions in the meristem and cell elongation in higher root zones, dependent or independent of auxin signaling, and thus affect PR growth (Ubeda-Tomás et al., 2012).

### LATERAL ROOTS

Lateral roots emerge post-embryonically from the PR and allow a plant to fully exploit the soil environment (Nibau et al., 2008). LR develop from three files of pericycle cells at the xylem pole (Beeckman et al., 2001; Casimiro et al., 2003). In these xylem pole founder cells, the cell cycle is re-activated and a lateral root primordium (LRP) initiates (De Smet, 2012). Priming of the founder cells takes place in the basal meristem, where oscillating auxin fluxes specify the cell fate (De Smet et al., 2007; Moreno-Risueno et al., 2010; Laskowski, 2013; Van Norman et al., 2013). Following priming, the cells undergo two asymmetric divisions that lead to a single-layered LRP. Subsequent periclinal divisions result in a dome-shaped LRP, which eventually emerges from the parental PR (Himanen et al., 2002; Benková and Bielach, 2010; Dastidar et al., 2012; Smith and De Smet, 2012). LR emergence is controlled by additional signaling factors, auxin import and export. Auxin transport affects both the patterning of the *de novo* formed LRP and of the cell layers overlaying the LRP to facilitate emergence (Swarup et al., 2008; Péret et al., 2009a, 2012, 2013).

Under normal growth conditions, LR initiation occurs acropetally, alternating along the PR. This positioning is determined by the auxin oscillation patterns in the basal meristem. Generally, there are more primed pericycle cells than LRP or LR initiated. This allows additional factors to regulate the position of a newly formed root branch. Both nutrients and internal factors, e.g., nitrate and sucrose affect LR formation and shape the root architecture (Malamy and Ryan, 2001; Malamy, 2005; Nibau et al., 2008; Petricka et al., 2012; Roycewicz and Malamy, 2012). Physical stimuli, such as mechanical obstruction and tropic responses also affect the spacing of LR (Richter et al., 2009; Lucas et al., 2013).

Lateral root formation is regulated by a complex interplay of molecular factors and different plant growth regulators (Nibau et al., 2008). Auxin-signaling has an important role from pericycle priming, initiation of cell division, and LRP formation to LR emergence (De Smet, 2012). In contrast to auxin, cytokinin inhibits LR development via a disruption of the patterning in *de novo* formed LRP and indirectly via an effect on polar auxin transport (Laplaze et al., 2007; Osmont et al., 2007; Růžička et al., 2009; Bielach et al., 2012). ABA acts as an auxin antagonist during LR initiation (Signora et al., 2001; De Smet et al., 2003; Fukaki and Tasaka, 2009; Guo et al., 2009). Besides the direct role in LR initiation, ABA mediates the balance between cytokinin and auxin and thus also indirectly inhibits LR formation (Shkolnik-Inbar and Bar-Zvi, 2010; Guo et al., 2012). Ethylene mediates auxin responses to inhibit LR formation (Růžička et al., 2007; Vandenbussche and Van Der Straeten, 2007; Negi et al., 2008, 2010; Lewis et al., 2011; Muday et al., 2012). Other hormones, such as GA, brassinosteroids, and strigolactones potentially act during LR formation via interference with auxin transport and sensitivity (Bao et al., 2004; Koltai et al., 2010; Kapulnik et al., 2011; Ruyter-Spira et al., 2011).

### ADVENTITIOUS ROOTS

Adventitious roots or shoot-borne roots, are roots that develop from non-root tissue, mostly aerial plant parts such as hypocotyls, leaves and stems. Developing adventitious root primordia (ARP) emerge from undifferentiated callus or from reprogrammed cells. Hence, the cellular origin of AR is uncertain and different tissues, often associated with the vasculature or cambial layers, are proposed to generate AR founder cells (Haissig, 1974; Davis and Haissig, 1994; Naija et al., 2008; Bellini et al., 2014). To gain insight into the cellular requirements for AR induction, several *Arabidopsis* explants have been used to analyze rooting: excised leaves, de-rooted plants, etiolated seedlings, light-grown seedlings, thin cell layers, and stem segments (Falasca and Altamura, 2003; Falasca et al., 2004; Ludwig-Müller et al., 2005; Fattorini et al., 2009; da Rocha Corrêa et al., 2012a,b; Sukumar et al., 2013; Verstraeten et al., 2013; Welander et al., 2014). In the *Arabidopsis* hypocotyl, AR originate from a cell layer reminiscent to the pericycle in the PR and therefore AR may share developmental characteristics with LR (Falasca and Altamura, 2003; Sorin et al., 2006; Li et al., 2009). In contrast to the stem-based *Arabidopsis* assays (Ludwig-Müller et al., 2005; Verstraeten et al., 2013; Welander et al., 2014), AR form spontaneously on etiolated hypocotyls upon transfer to light (Sorin et al., 2005). Even though AR induction on the hypocotyl does not require exogenous auxin or wounding, both factors enhance AR formation. For example, AR initiate on the hypocotyl of light-grown seedlings from which the PR is excised (Sukumar et al., 2013). In this latter system, wounding acts as the external trigger for *de novo* ARP formation. The availability of the two *Arabidopsis* hypocotyl systems allows future comparisons to decipher the role of wounding as a requirement to initiate AR.

Adventitious root formation is a complex process, influenced by multiple factors, including phytohormones, light, wounding, and stress. Auxin plays a central role (De Klerk et al., 1999; Pop et al., 2011) and cytokinin acts as an auxin antagonist (Kuroha and Satoh, 2007; Della Rovere et al., 2013). However, other plant growth regulators, such as ethylene, jasmonic acid, and strigolactones have specific effects on AR and will be discussed below.

## KEY ROLE OF AUXIN IN ROOT INDUCTION AND DEVELOPMENT

Auxin is important for many aspects of root development, including initiation and emergence, patterning of apical meristem, gravitropism, and root elongation. Auxin plays a key role in both AR and LR development (De Klerk et al., 1999; Overvoorde et al., 2010; Pop et al., 2011; Lavenus et al., 2013; Bellini et al., 2014). Mutants or treatments resulting in disturbed polar auxin transport show rooting defects, both in LR and AR formation (Reed et al., 1998; Bhalerao et al., 2002; Himanen et al., 2002; Li et al., 2012; Sukumar et al., 2013). While auxins and auxin signaling are essential for all stages of LR development (Péret et al., 2009a, 2013; Dastidar et al., 2012; Lavenus et al., 2013), exogenous auxin is only stimulating during the first stages of AR development and inhibits later developmental stages (De Klerk et al., 1995, 1999; Bellamine et al., 1998). This indicates that both root-types have a different sensitivity to exogenous auxin. Besides exogenous or newly biosynthesized auxin, auxin transport, in particular via the ABC-type multi-drug-resistance ABCB19 transporter, is also essential for AR induction in hypocotyls (Sukumar et al., 2013). The release of auxin from storage forms such as indole-3-butyric acid (IBA) or IAA-conjugates also contributes to organ formation. In recent studies using a synthetic auxin analog naxillin, root cap specific IBA-conversion was shown to control priming of pericycle cells and to determine the positioning of LR (De Rybel et al., 2012; Schlicht et al., 2013). Since both AR and LR development depend on auxin signaling factors, exogenous auxin application does not allow root-type specific research.

## MOLECULAR FACTORS INVOLVED IN THE INITIATION OF AR IN THE HYPOCOTYL VERSUS LR FORMATION IN THE PR

Lateral root organogenesis depends on auxin signaling and dominant mutants of the *solitary root SLR/IAA14* signal transduction factor lead to omission of cell divisions in the pericycle and aborted LR formation (Fukaki et al., 2002; Vanneste et al., 2005). This indicates a direct link between auxin signaling and formative cell divisions. LR initiation starts with a unique process called priming, which determines the pericycle cells that engage to form LR (Van Norman et al., 2013). Priming is only possible in tip-growing organs like roots, where events at the tip determine later responses of the continuously developing cell lines so that the root can adapt to its environment. Priming is mainly controlled by *IAA28*, with *IAA8* and *IAA19* in a redundant role. Priming takes place in the basal meristem, where oscillating auxin fluxes specify LR founder cells in the pericycle (De Smet et al., 2007; Moreno-Risueno et al., 2010). Downstream of *IAA28* the priming events are controlled by *ARF5*, *6*, *8,* and *19* (Lavenus et al., 2013). An additional auxin response factor, *ARF7*, shows an oscillating expression pattern in the pericycle, which suggest interaction between *IAA28* and *ARF7* A recently identified GATA-type transcription factor, *GATA23*, is specifically expressed in the primed pericycle cells, even before the occurrence of asymmetric divisions (De Rybel et al., 2010; Yadav et al., 2010; Dastidar et al., 2012). GATA23-expression depends on *IAA28*, *ARF7,* and *ARF19* (De Rybel et al., 2010; Yadav et al., 2010). *ARF6-ARF8*-mediated signaling with GATA23 as a target, specifies LR founder cell (Lavenus et al., 2013). After the priming, asymmetric divisions generate *de novo* LRP. These asymmetric divisions are controlled by *Arabidopsis crinkly 4 (ACR4;* De Smet et al., 2008). During the subsequent events in LR development, *SLR/IAA14*, *ARF7* and *ARF19* interact, and the *Lateral Organ Boundaries-domain/Asymmetric Leaves2-like (LBD/ASL)* family positively regulates LR formation in an auxin dependent way (Okushima et al., 2007). Downstream of the *SLR/ARF7,19* signaling module, *SHY2/IAA3* regulates further LR development (Goh et al., 2012a,b; Lavenus et al., 2013). Via PIN-mediated auxin transport, auxin accumulates at the tip of a developing primordium. This results in the activation of genes necessary for *de novo* LRP patterning (Péret et al., 2009a). Auxin also affects the cells overlaying the LRP to facilitate emergence. The auxin-import carrier LAX3 regulates the expression of cell wall remodeling enzymes in the outer root tissues and auxin induces aquaporins to facilitate root emergence (Péret et al., 2009a, 2012). In general, at all different stages of LR development, different auxin signaling components play important roles and determine organogenic responses. Recently cell to cell communication via CLE-peptides or *ACR4* is shown to be involved in LR organogenesis and programming of the LRP neighboring cells (Stahl and Simon, 2012; Delay et al., 2013; Fernandez et al., 2013; Yue and Beeckman, 2014).

In the *Arabidopsis* hypocotyl, AR develop from the pericycle (Boerjan et al., 1995; Sorin et al., 2005). In this cell layer, there are so far, no evidence for priming events. The cell number of a hypocotyl is fixed during embryogenesis and no further cell divisions occur during hypocotyl growth. Instead, hypocotyls show a determinate growth pattern with most of their expansions resulting from cell elongation (Gendreau et al., 1997). This is in contrast to the indeterminately growing PR, which has the capacity to determine new sites for LRP formation while it extends into the environment. The final length of the hypocotyl mainly depends on environmental factors such as light and hormones, including auxin, GA, and brassinosteroids (Jensen et al., 1998; Cowling and Harberd, 1999; De Grauwe et al., 2005; Vandenbussche et al., 2005; Chapman et al., 2012). As most hypocotyls cells are present already in the embryo stage, AR priming likely depends on environmental factors. In this context it is of interest to analyze specific molecular markers associated with LR-priming in hypocotyls exposed to different growth conditions.

Similar to LR development, AR development is controlled by auxin-dependent signaling processes. In the hypocotyl, *SLR/IAA14* also acts as a factor determining root formation capacity and in *slr* mutants, no ARP get formed (Fukaki et al., 2002). In the intact hypocotyl, light-induction is necessary to induce AR. This process is mediated by *LONG HYPOCOTYL (HY5)* and *argonaute 1* (Sorin et al., 2005, 2006; Sibout et al., 2006). Proteomic analysis in hypocotyls of the AR-defective *ago1* mutant showed that auxin-conjugate levels are reduced and that *AUXIN RESPONSE FACTOR 17 (ARF17)* is overexpressed. This suggests *ARF17* has a negative role during AR formation (Sorin et al., 2005, 2006). Additionally, auxin-inducible Gretchen Hagen 3-like proteins: GH3-3, GH3-5, and GH3-6 fulfill a role in the fine-tuning of AR initiation via de modulation of jasmonate-homeostasis (Gutierrez et al., 2012). Because *ARF17* is a target of miRNA160, a selection of other *ARFs* regulated by miRNAs were investigated and AR numbers were recorded in *ARF6*, *8*, *10,* and *16* mutants. *Arf6-3* and *arf8-1* developed less AR, which indicates that *ARF6* and *ARF8* act as positive regulators of AR formation. *ARF6, ARF8,* and *ARF17* have overlapping expression profiles and regulate each other’s transcription level. Moreover, the balance between repressing (*ARF17*) and activating (*ARF6* and *ARF8*) factors is post-transcriptionally regulated by miR160 and miR167 (Gutierrez et al., 2009). The regulation of JA-homeostasis by GH3 enzymes is sufficient to reduce active jasmonate (JA), an inhibitor of adventitious rooting, and to induce AR (Gutierrez et al., 2012).

The common role of *SLR/IAA14* and the identification of similar signal transduction factors (*ARF6*, *ARF8*) indicates similarities between the auxin-mediated signaling pathway controlling AR development and the LR induction pathway. Moreover, *gh3* mutants are not specific to AR processes only and show small enhancement of LR formation because the enzymes they encode are also involved in auxin homeostasis (Staswick et al., 2005; Zhang et al., 2007; Gutierrez et al., 2012). Other genes, such as *LRP1* are expressed in both LRP and ARP and do hence not differentiate between root-types (Smith and Fedoroff, 1995). On the other hand, *ARF17* and *AGO1* are uniquely involved in AR organogenesis, which suggests a separate set of regulators exists (Sorin et al., 2005; Gutierrez et al., 2009, 2012; Bellini et al., 2014). Further support for an independent regulation of AR and LR is the *Arabidopsis monopteros* mutant, which does not form a functional PR but does develop AR (Przemeck et al., 1996; Bellini et al., 2014). Hence, LR and AR share certain signaling elements but also depend on root-type specific signaling components. In addition to unique auxin-dependent regulators also cytokinin specifically controls either root-type. The WOODEN LEG *wol3* mutants, defective for the cytokinin receptor *ARABIDOPSIS HISTIDINE KINASE 4 (AHK4)*, shows defects of vascular development in the PR and LR but not in *de novo* initiated AR (Kuroha et al., 2006; Kuroha and Satoh, 2007). Also the *A. thaliana pectin methylesterase 3* gene has a specific role during AR development. This gene is expressed in vascular tissues and mutants do not show defects in LR but develop more AR (Guénin et al., 2011). As vascular development is a common nominator between the latter genes, regulators of vascular development might differentiate between different root-types. Some of the temperature-sensitive *Arabidopsis* mutants identified by Konishi and Sugiyama (2003), are specifically affected in AR development. For example the *root initiation defective1* (*rid1*) mutant fails to initiate ARP in the hypocotyl, but still initiates LR in PR segments (Konishi and Sugiyama, 2003). Specifc cell-wall-remodeling proteins are important for LR development, without having a role during AR initiation (Lewis et al., 2013). Auxin transport is important for both root-types, but whereas PIN proteins are important during LR development (Benková et al., 2003; Laskowski et al., 2008; Lewis et al., 2011; Marhavy et al., 2013), ABCB19 has a more pivotal role in hypocotyl-based AR formation (Sukumar et al., 2013).

## HORMONES WITH DIFFERENTIAL ROLE IN PR BRANCHING OR HYPOCOTYL AR

Auxin can been seen as the main regulator of rooting competence, but other plant growth regulators might contribute to the fine-tuning of the rooting response or they act during the steps at which the root identity is obtained.

The volatile plant growth regulator, ethylene, is probably the hormone that differentiates most between root-types. In many species, ethylene is found to have a positive effect on AR development and emergence. For example in mung bean or sunflower hypocotyl cuttings, prolonged exposure to exogenous 1-aminocyclopropane-1-carboxylic acid (ACC), the precursor of ethylene, results in increased root numbers (Jusaitis, 1986; Liu et al., 1990; Pan et al., 2002). Also in other species, such as *Rumex* and the monocots maize and rice, exogenous ethylene has a positive effect on AR formation (Drew et al., 1979, 1989). In both *Arabidopsis* and tomato mutants that overproduce ethylene or have a constitutive response to ethylene (resp. *eto1, ctr1,* and *epi*), lower numbers of LR are recorded, while in the ethylene-insensitive mutants (*ein2* and *Nr*) more root branches are formed (Negi et al., 2008, 2010). However, in tomato hypocotyls the function of ethylene is opposite and ethylene promotes the formation of AR (Clark et al., 1999; Negi et al., 2010). In *A. thaliana* however, this stimulatory role of ethylene on AR formation is not observed. In *Petunia*, reduced AR formation is observed in ethylene-insensitive plants (Clark et al., 1999). It often is suggested that ethylene is needed to elicit rhizogenesis in the presence of auxin and probably functions through interaction with auxin sensitivity and transport (Riov and Yang, 1989; Růžička et al., 2007; Negi et al., 2010; Strader et al., 2010; Lewis et al., 2011). However, whereas ethylene and auxin interact to inhibit root growth, ethylene acts independent of auxin signaling in etiolated *Arabidopsis* hypocotyls (Stepanova et al., 2007). Consequently we conclude that different tissues have a different sensitivity to the same growth regulator and the tissue thus determines the organogenic responses.

The contrasting effects of ethylene in *Arabidopsis* and tomato may reflect a differential adaptation to flooding. Tomato is sensitive to water-logging and moisture, and the formation of AR is started shortly after submergence (McNamara and Mitchell, 1991). The submergence of the hypocotyl of tomato seedlings and the subsequent oxygen shortage, stimulates ethylene production and induces hypocotyl-based AR formation (Vidoz et al., 2010). Also in sunflower and *Rumex*, water-logging results in AR formation, stimulated by ethylene (Wample and Reid, 1979; Visser et al., 1996a,b). In rice, ethylene is important for the induction of epidermal cell death to facilitate AR emergence, a process that is flooding- and anoxia-dependent (Mergermann and Sauter, 2000; Steffens et al., 2006). In these cases, ethylene modulates auxin transport and the resulting auxin accumulation stimulates AR formation. Besides its role in flooding and anoxia, ethylene also functions in wounding responses, which may be linked with AR formation in cuttings (O’Donnell et al., 1996). Moreover, ethylene does not only interact with auxin, but the complex interactions between ethylene, GA and ABA in deepwater rice illustrates the importance of finely regulated hormone interactions during AR formation (Steffens et al., 2006).

Jasmonates are stress-related hormones that are rapidly synthesized upon wounding but also are involved in developmental processes (Creelman et al., 1992; Glauser et al., 2008; Koo et al., 2009). Methyl jasmonate (MeJA) is shown to inhibit PR growth, while it promotes LR formation in *Arabidopsis* and rice (Raya-Gonzalez et al., 2012; Hsu et al., 2013). In the JA receptor mutant *coi1*, LR-positioning is disturbed and JA acts both through an auxin-dependent and an auxin-independent pathway (Raya-Gonzalez et al., 2012). MeJA stimulates AR initiation in tobacco thin cell layers (Fattorini et al., 2009). In stem cuttings of *Petunia* however, JA accumulating at the base and inhibits AR emergence (Ahkami et al., 2009). In the *Arabidopsis* hypocotyl, JA negatively regulates AR development (Gutierrez et al., 2012). JA therefore shows opposing effects on root formation depending on the plant organ: hypocotyl, stem, or root.

Both strigolactones and GA have an inhibitory role during AR and LR induction. In the presence of the synthetic strigolactone analogs, GR24 or CISA, AR formation is strongly reduced, whereas inhibition of LR development much less pronounced (Koltai et al., 2010; Kapulnik et al., 2011; Ruyter-Spira et al., 2011; Rasmussen et al., 2012, 2013; Brewer et al., 2013). GA treatment in *Arabidopsis* hypocotyls inhibits adventitious rooting (Mauriat et al., 2014). This is in line with earlier reports in which ectopic increase of GA production in the stem of tobacco and rice was shown to reduce AR induction (Lo et al., 2008; Niu et al., 2013). Similarly, GA-deficient and GA-insensitive transgenic *Populus,* show increased LR proliferation (Gou et al., 2010). GA primarily reduces polar auxin transport, thereby limiting the auxin availability for the induction of cell divisions (Gou et al., 2010; Niu et al., 2013; Mauriat et al., 2014). A more specialized regulation of root induction by GA is observed in deep water rice, where GA promotes AR initiation through interactions with ethylene (Steffens et al., 2006). The stress hormone ABA, negatively regulates LR initiation and development (Signora et al., 2001; De Smet et al., 2003). Despite the role of ABA in wound responses, the establishment of a sink and QC development, the role of ABA in AR development has not been investigated in detail yet. Moreover, contradicting findings about positive and negative effects are reported. This indicates that subtle differences in the plant state or in the interactions with other plant growth regulators affect the outcome of ABA-signaling. Cytokinins counteract the root-stimulating effect of auxin in both LR and AR organogenesis, are important for the establishment of a functional meristem in both root-types and therefore have no root-type differential role (Della Rovere et al., 2013).

In addition to hormonal factors, also signaling factors, such as nitric oxide (NO) determine the rooting responses. NO is related to stress responses and NO-production is enhanced under elevated CO_2_ conditions and after wounding (Wang et al., 2013). NO is an essential factor to determine the rooting potential of juvenile cuttings (Abu-Abied et al., 2012). In eucalypt, high NO-concentrations are correlated with a higher capacity to form AR, but in *Arabidopsis* constitutive expression of nitrate reductase does not affect the formation of either LR or AR (Abu-Abied et al., 2012). Moreover, NO and cyclic GMP function as messengers acting downstream of auxin during AR formation in cucumber hypocotyls (Pagnussat et al., 2003, 2004). NO regulates cell divisions and organogenic processes in a calcium-dependent way (Lanteri et al., 2006). NO is emerging as a player in LR development because local, peroxisomal IBA to IAA conversion is important for LR formation and NO acts as a signal during this process (Mendez-Bravo et al., 2010; De Rybel et al., 2012; Schlicht et al., 2013). Moreover, NO interacts with other hormone signaling pathways (Simontacchi et al., 2013). NO-homeostasis is regulated by the plant hormone cytokinin and direct interaction between NO and cytokinin suppresses the action of NO (Liu et al., 2013). This correlates with the negative action of NO during AR development and the inhibitory role of cytokinin on AR formation is at least partly mediated through NO. Polyamines and hydrogen peroxide are other important signaling molecules in AR development (Nag et al., 2001; Couee et al., 2004; Bai et al., 2007; Kevers et al., 2009; Liao et al., 2009; Ragonezi et al., 2010). Polyamines regulate cell divisions, the sensitivity to endogenous auxin, differentiation and regulate ethylene-biosynthesis to translate environmental changes into root development (Couee et al., 2004; Naija et al., 2008).

## ENVIRONMENTAL FACTORS WITH DIFFERENTIAL EFFECT ON AR AND LR FORMATION

The different role of plant growth regulators and signaling factors, requires a tight control of initiation and emergence fine-tuning during the development of different root-types. As hormones are translators of environmental conditions, the above mentioned differences most likely represent environmental conditions that differentiate between AR and LR formation (Rubio et al., 2009). The plant root system is responsible for the acquisition of water and nutrients from soil and it is embedded in a complex environment with biotic and abiotic interactions. It is therefore not surprising that the root system architecture is highly influenced by environmental signals, such as water availability and nutrient concentration (Malamy, 2005; Monshausen and Gilroy, 2009). Different root-types flexibly respond to the environmental conditions and generate a root system that allows plants to perform well in their growth conditions (Hodge, 2004; Jones and Ljung, 2012).

Whereas in some species, AR initiate spontaneously, in other species AR are only formed upon stresses such as wounding or flooding or induced by nutrient shortage. Both in plant cuttings and in intact plants, wounding-associated dedifferentiation is often a requirement to form roots (da Costa et al., 2013). Upon PR damage or excision of cuttings, wound-induced AR assist the plant’s survival. Upon wounding, auxin accumulates rapidly at the base of a segment and stimulates cell divisions in this region (De Klerk et al., 1999). Auxin accumulation also stimulates the creation of a carbohydrate sink, which is an additional stimulants for AR development (Agulló-Antón et al., 2011). In *Arabidopsis*, removal of the PR leads to rapid AR induction at the hypocotyl base (Sukumar et al., 2013), but AR formation also takes place without wounding in etiolated hypocotyls (Sorin et al., 2005).

A general tendency is that roots branch more in nutrient-rich conditions (Robinson, 1994; López-Bucio et al., 2003; Hodge, 2004; Krouk et al., 2011). High nitrate inhibits LR development, while low nitrate has no effect on PR growth and promotes LR initiation (Robinson, 1994; Zhang et al., 1999; Signora et al., 2001). ABA mediates these nitrate effects (Signora et al., 2001; De Smet et al., 2003, 2006b). The direct relation between nitrogen-availability and AR development still needs to be evaluated, but in horticultural species, such as in rose, addition of nitrate or ammonia does not affect AR formation (Hyndman et al., 1981). Nitrogen also affects the formation of aerenchyma in AR and thereby improves the oxygen exchange (Drew et al., 1989; He et al., 1992). Phosphorous on the other hand has a different effect on different root-types. Due to the poor mobility in the soil, phosphorous often is a limiting growth factor (Abel, 2011). Plants optimize their development to maximize phosphate usage by branching and the formation of AR in the top soil stratum. Low phosphate does not affect basal PR growth or LR induction, but it preferentially stimulates newly initiated AR on the hypocotyl and lower stems (Lynch and Brown, 2001; Williamson et al., 2001; Walk et al., 2006). Possibly, the phosphate responses occur via interaction with auxin perception, signaling and redistribution (López-Bucio et al., 2002, 2005; Al-Ghazi et al., 2003; Nacry et al., 2005; Pérez-Torres et al., 2008; Jones and Ljung, 2012). During phosphate deficiency, strigolactones are important and their biosynthesis is directly regulated by the presence of phosphorous (López-Ráez et al., 2008; Yoneyama et al., 2012; Foo et al., 2013). Strigolactones have both a direct and an indirect effect on root responses, via the induction of the auxin receptor *TIR1* (Kapulnik et al., 2011; Ruyter-Spira et al., 2011; Mayzlish-Gati et al., 2012). In general, AR are considered beneficial to sustain shoot growth and the formation of AR is more energy-efficient than other root-types depending on the nutrient-availability. Thanks to the presence of aerenchyma, which enhances gas exchange, AR formation has advantages for the plant (Miller et al., 2003; Ochoa et al., 2006). However, research describing AR development in nutrient-deprived conditions is limited.

Besides nutrients, also water is essential for plant growth. To increase water-use efficiency in water-limiting conditions, PR elongation is promoted to reach deeper water resources (Garay-Arroyo et al., 2012; Jones and Ljung, 2012; Xu et al., 2013). ABA translates the soil water status into root development. In addition to the effect on the PR elongation, LR initiation and development are inhibited by drought through interactions of ABA, auxin and cytokinin (Signora et al., 2001; De Smet et al., 2003, 2006b; Malamy, 2005; Shkolnik-Inbar and Bar-Zvi, 2010; Xu et al., 2013). Prolonged drought stress results in the development of a short PR and dormant LR, which rapidly resume growth upon rehydration (Vartanian et al., 1994; Zolla et al., 2010; Sreenivasulu et al., 2012). Similarly, AR development could be inhibited by drought, but research about the effect of drought on AR development or the involvement of ABA in the regulation of AR formation is limited. Submergence or flooding on the other hand, induce AR to replace the basal root system (Voesenek et al., 1996; Mergermann and Sauter, 2000; Ahmed et al., 2013; Sauter, 2013). The main reason for this response is the lack of oxygen, which hampers nutrient- and water-uptake in the PR (Bailey-Serres and Voesenek, 2008; Calvo-Polanco et al., 2012) and as an adaptation, stem- or node-borne AR are formed to replace soil-borne roots (Vidoz et al., 2010). The metabolic costs to maintain the PR are higher than the costs to form AR, so the initiation of AR is a logical trade-off for the root system (Miller et al., 2003; Walk et al., 2006). Upon submergence, plant species adopt two strategies: either *de novo* formation of ARP [e.g., in tomato and sunflower (Wample and Reid, 1976, 1979; Negi et al., 2010)] or emergence of dormant ARP, such as in *Poaceae* (Malamy, 2005; Steffens et al., 2006). Ethylene acts in both strategies and increase auxin transport and the sensitivity to auxin in the rooting zones, thereby promoting the formation of AR and the emergence of pre-formed root initials. AR grow horizontally with more access to oxygen and contain aerenchyma, in which gas exchange occurs efficiently (Gaxiola et al., 2010; Abiko et al., 2012; Ahmed et al., 2013). Besides the supply of oxygen, AR have higher hydraulic conductivity and thus supply the plants with sufficient water to sustain development (Calvo-Polanco et al., 2012).

## CONCLUSION

**FIGURE 1:**
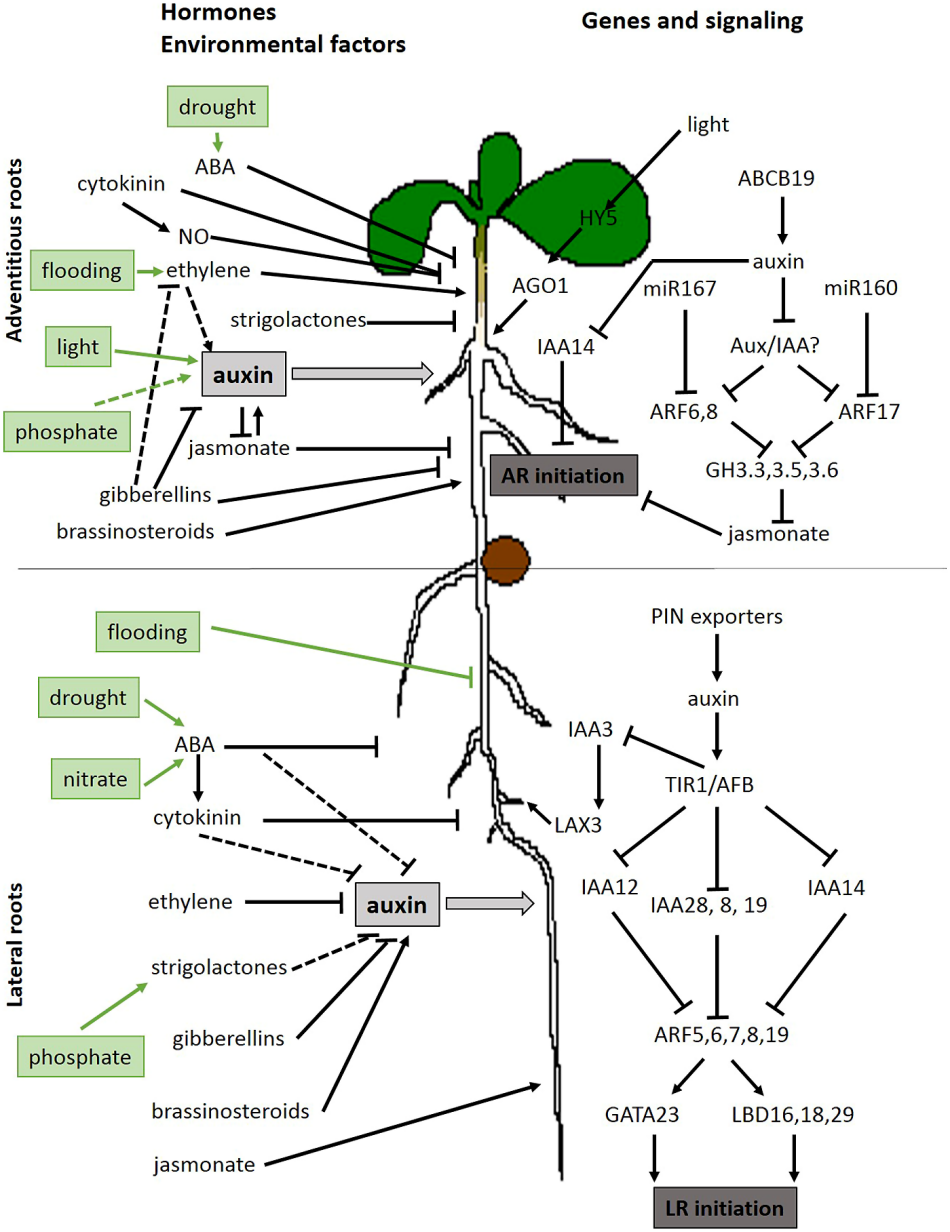
**Overview of hormonal, environmental, and genetic factors involved in adventitious roots developing from the hypocotyl and lateral root initiation on the primary root.** Dashed lines represent possible interactions. Full lines represent published interactions (references throughout this article). Positive interactions are indicated with a sharp arrowhead and negative interactions with a blunt arrowhead.

In *A. thaliana* the PR, LR, and AR together form a flexible root system that is capable to adapt to changing environmental conditions. Despite the complex mix of different root-types, physiological and genetic studies have started to pinpoint specific factors to determine root identity and to control the development of one root-type over another. We believe that LR and AR share pathways, but that both root-types also have a specific set of signaling components. Because both root-types have functional similarities and differences, the different pathways intertwine at certain positions. Although some signaling factors are shared, additional research is required to draw the complex map of all interacting factors (da Costa et al., 2013). On the other hand, other factors uniquely regulate one specific root-type and help to control the investment of a plant in either the PR system or secondary AR. These factors are both genetic, hormonal and environmental, and also the plant’s endogenous conditions affects the rooting capacity. An overview of the hormonal, environmental and genetic factors involved in the generation of lateral or (ARs) is given in **Figure 1**. A difference in tissue origin affects the regulation of the organogenic response and the conditions in the hypocotyl pericycle differ from those in a similar cell layer in the PR. Moreover, also the formation of stem-based AR is regulated by different factors than hypocotyl AR (Verstraeten et al., 2013; Welander et al., 2014). We only just started to decipher differences in PR, LR, and AR root development and more research will lead to a better understanding of the different root-types and treatments to optimize plant growth in all conditions.

## Conflict of Interest Statement

The authors declare that the research was conducted in the absence of any commercial or financial relationships that could be construed as a potential conflict of interest.
